# A comparison of spatial clustering and cluster detection techniques for childhood leukemia incidence in Ohio, 1996 – 2003

**DOI:** 10.1186/1476-072X-6-13

**Published:** 2007-03-27

**Authors:** David C Wheeler

**Affiliations:** 1 Department of Biostatistics, Emory University, Atlanta, GA, USA

## Abstract

**Background:**

Spatial cluster detection is an important tool in cancer surveillance to identify areas of elevated risk and to generate hypotheses about cancer etiology. There are many cluster detection methods used in spatial epidemiology to investigate suspicious groupings of cancer occurrences in regional count data and case-control data, where controls are sampled from the at-risk population. Numerous studies in the literature have focused on childhood leukemia because of its relatively large incidence among children compared with other malignant diseases and substantial public concern over elevated leukemia incidence. The main focus of this paper is an analysis of the spatial distribution of leukemia incidence among children from 0 to 14 years of age in Ohio from 1996–2003 using individual case data from the Ohio Cancer Incidence Surveillance System (OCISS).

Specifically, we explore whether there is statistically significant global clustering and if there are statistically significant local clusters of individual leukemia cases in Ohio using numerous published methods of spatial cluster detection, including spatial point process summary methods, a nearest neighbor method, and a local rate scanning method. We use the *K *function, Cuzick and Edward's method, and the kernel intensity function to test for significant global clustering and the kernel intensity function and Kulldorff's spatial scan statistic in SaTScan to test for significant local clusters.

**Results:**

We found some evidence, although inconclusive, of significant local clusters in childhood leukemia in Ohio, but no significant overall clustering. The findings from the local cluster detection analyses are not consistent for the different cluster detection techniques, where the spatial scan method in SaTScan does not find statistically significant local clusters, while the kernel intensity function method suggests statistically significant clusters in areas of central, southern, and eastern Ohio. The findings are consistent for the different tests of global clustering, where no significant clustering is demonstrated with any of the techniques when all age cases are considered together.

**Conclusion:**

This comparative study for childhood leukemia clustering and clusters in Ohio revealed several research issues in practical spatial cluster detection. Among them, flexibility in cluster shape detection should be an issue for consideration.

## Background

Spatial cluster detection is an important tool in cancer surveillance to identify areas of elevated risk and to generate subsequent hypotheses about cancer etiology. A spatial disease cluster may be defined as an area with an unusually elevated disease incidence rate [1,2]. There are several cluster detection methods used in spatial epidemiology to investigate apparently suspicious groupings of cancer occurrences in both regional count data and case-control data, where the controls are often sampled from the at-risk population and are used to estimate local relative risk or local rates, depending on the method utilized. Numerous studies [3,4] in the literature have focused on childhood leukemia because of its relatively large incidence among children compared with other malignant diseases, its apparent tendency to cluster, and the substantial public concern over locally elevated leukemia incidence. Many cluster-inducing factors have been considered in the literature on leukemia, including infectious agents [5] and population mixing[6,7], environmental pollution [8], such as benzene [9], pesticides [10], and radiation [11], and geographic variation in other risk factors, such as inherited genetic risk [12], maternal alcohol consumption and cigarette smoking [13], and socioeconomic status [14]. There are many studies of potential cancer clusters in the literature, and the reader is referred to two useful reviews [15,16].

In this paper, we present an empirical analysis of the spatial distribution of leukemia incidence among children from 0 to 14 years of age in Ohio from 1996–2003 using individual case data from the Ohio Cancer Incidence Surveillance System (OCISS) in response to public concern of potentially elevated cancer risk among children in areas of Ohio. There has been no previous comprehensive and systematic spatial analysis of potential clustering of childhood leukemia in Ohio. Other studies [7,17] of potential clusters of childhood leukemia in Ohio do not include spatial analysis methods or individual case data, and instead typically use chi-square tests of differences in expected and observed case counts in census or political units. This approach is not expressly a test for clustering or clusters, but a test of elevated counts inside an often heterogeneously populated area, for example, a county, and the test for one area is considered independently of other areas. This approach does not consider if areas with significantly more cases than expected are spatially juxtaposed [18,19]. We choose not to use aggregated case data at the census level because we have access to individual case and control data, want to avoid unstable regional rates caused by small observed case counts and small population counts [20,21], and want to avoid the modifiable areal unit problem (MAUP) [19] arising from using political boundaries that are arbitrarily related to public health. More specifically, we explore whether there is or is not statistically significant global clustering and local clusters of individual leukemia cases using numerous published methods of spatial cluster detection. We, therefore, address the questions of whether childhood leukemia cases have a significant tendency to cluster in Ohio and where the most unusual groupings of cases, if any, are located. The evaluation of the null hypothesis of no significant global spatial clustering of childhood leukemia uses three different methods: the *K *function, the kernel intensity function, and Cuzick and Edwards' method. See Waller and Jacquez [22] for a discussion of hypotheses in tests for disease clustering. We evaluate the null hypothesis of no local areas of elevated childhood leukemia risk using the kernel intensity function and Kulldorff's scan statistic. The distinction between clustering and cluster detection tests has been made in the literature [1,19,23-25], and we follow that distinction in this paper. Clustering and cluster detection tests are viewed as complimentary, as they test different hypotheses. A simulation study by Waller et al. [1] indicated that it is possible to have a significant cluster, but no overall significant clustering. In spatial point processes, the first-order property (intensity function) of the process is used for a test of clusters and the second-order property (K function) is used as a test for global clustering [19].

Our comparison of cluster detection methods is similar in spirit to Griffith's comparison of disease mapping techniques for West Nile Virus [26], and is motivated by the numerous and diverse analytical options currently available to cancer prevention researchers investigating potential clusters with case-control data. There have been methodological comparison papers in the literature for spatial cluster detection [27-31], but none exclusively for individual level data. Our selection set of methods to compare in this paper includes the leading published methods designed for individual level case data that are currently implemented in publicly available software. We use R software [32] to implement the *K *function and kernel intensity function, ClusterSeer software [33] for Cuzick and Edwards' method, and SaTScan [34] for Kulldorff's scan statistic. The reader interested in a comparison of general functionality of free software that may be used for cluster analysis is referred to a review by Anselin [35], although not all features compared in the review are expressly for individual case data. We next briefly review each of the clustering and cluster detection techniques and then present and compare the findings from them.

## Methods

### Data

**Figure 1:**
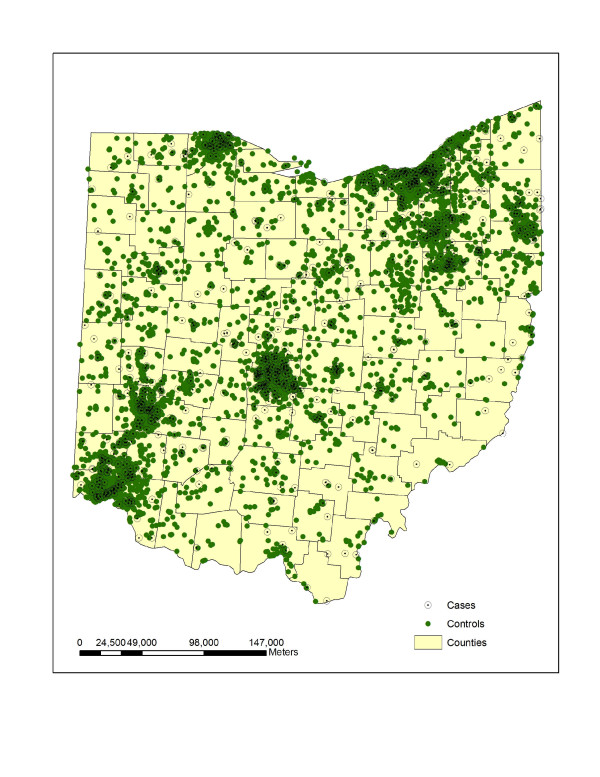
Childhood leukemia cases (unfilled circles) and controls (filled circles) for years 1996–2003.

In the subsequent analysis, we use 738 individual OCISS cases diagnosed between 1996–2003, geocoded to the street level using geographic information system (GIS) software from ESRI [36]. The use of the cancer data in this study was approved by the Ohio Department of Health Institutional Review Board. The childhood (0–14) leukemia rate for Ohio between years 1996–2003 was 4.2 per 100,000 persons, compared to the SEER rate of 4.8 per 100,000 persons [37]. The completeness of incidence data in OCISS varies by year, for example, the percent of completeness was 85% in 1996, 92% in 1998, and 95% in 1999 [38]. We excluded cases from the analysis that were not address matched to the street level and were matched only to the ZIP Code centroid level. There were 86 cases that were matched to the centroid level and omitted to avoid inducing spurious clustering. A map of these cases showed an essentially random pattern across Ohio, neither occurring in exclusively urban or rural areas, and the lack of pattern or concentration in the cases helped to justify removing them from the study. As stated earlier, this paper focuses on a spatial case-control study, which requires controls sampled from the at-risk population for leukemia that did not develop leukemia during the same time period of births as the reported cases. We used as controls births sampled from the Ohio Vital Statistics (OVS) records where there were digital files available, from 1989–2003, which contains most of the possible birth years of cases (1982–2003). More specifically, we began with 21,906 randomly sampled birth records from OVS that were geocoded to the street level and then systematically sampled 7,302 records as controls, selecting every third record where the birth records were ordered by longitude and latitude. Presumably, any rural bias in the failure to locate addresses in the geocoding process would affect both cases and controls, so any impact in the analysis presented here is likely slight. The systematic sampling scheme was employed to provide a geographically representative sample of the at-risk population and resulted in a control-case ratio of approximately 10 to 1. Visual comparison of the controls and the larger set of birth records suggested the controls were a spatially representative sample. The control-case ratio used was a compromise between using as many controls as possible and computation considerations for certain methods. The idea of using as many controls as possible draws from Peter Diggle's comments in his written discussion of Cuzick and Edwards' paper [39] introducing their nearest neighbor test for clustering. In fact, in a preliminary analysis with the Cuzick and Edwards method we used a control-case ratio of 3 to 1 to align with traditional case-control studies in epidemiology, but found significant clustering at small distances that appeared to be due to a lack of an adequate number of controls in some rural areas. A visual display of the controls using this ratio suggested that controls underrepresented the at-risk population in some rural areas. The ideal number of controls to use relative to the number of observed cases and the underlying population structure is an important issue left for future research. A map of the sampled controls from a 10 to 1 ratio of controls to cases shows a pattern that appears to better approximate the general distribution of population in Ohio. Figure 1 displays the sampled controls as filled circles and the cases as open circles, where points have been uniformly randomly shifted from their true locations for data confidentiality [40]. Based on the figure, it appears there is no clear overall clustering in the cases and no obvious clusters of cases, after visually accounting for the distribution of population, as represented by the controls. However, the map of cases can be misleading because of the potential for many cases to be located at nearly the same location given the map scale, and a statistical analysis is needed to formally test for clustering and the presence and location of clusters. To investigate potential clustering and local clusters, we assume a realization of a heterogeneous Poisson point process for the controls and a second such process for the cases, with a constant risk null hypothesis where more cases are expected with a larger population at risk. To test for spatial heterogeneity in leukemia risk among groups, we perform four total sub-analyses, one for cases of acute lymphocytic leukemia (ALL), the dominant sub-type of leukemia among children, and three for mutually exclusive age groups of 0–4, 5–9, and 10–14 with the Cuzick and Edwards method and the scan statistic in SaTScan.

## Results

### *K *function

**Figure 2:**
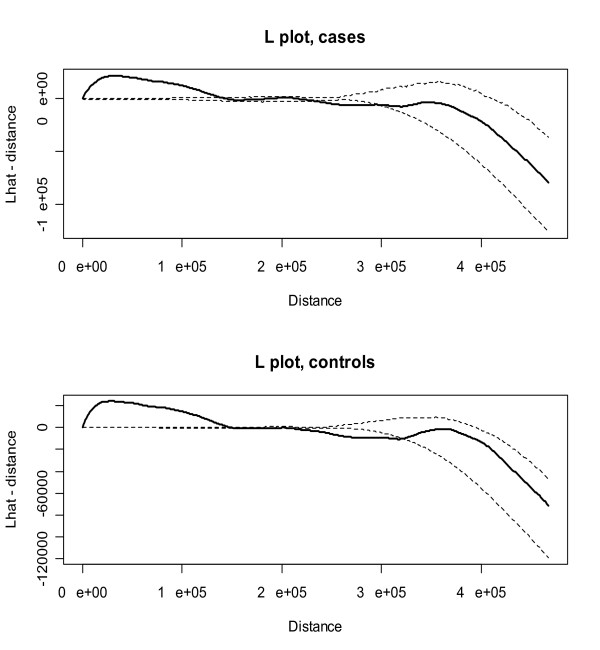
*K *functions (solid) for cases and controls with confidence bands (dashed) and distance in meters.

The *K *function is a method introduced by Ripley [41] for testing for general clustering in a point pattern. It measures how many events occur within a certain distance of other events. A simple formula for the *K *function is *K*(*h*) = (average number of events within distance *h *of a randomly chosen event)/(average number of events per unit area). Also see Diggle [42] and Waller and Gotway [19] for a detailed discussion of the *K *function. The *K *function uses a vector of distances ***h ***to calculate the function many times at a range of distances in the study area. One can calculate a transformation, L^
 MathType@MTEF@5@5@+=feaafiart1ev1aaatCvAUfKttLearuWrP9MDH5MBPbIqV92AaeXatLxBI9gBaebbnrfifHhDYfgasaacH8akY=wiFfYdH8Gipec8Eeeu0xXdbba9frFj0=OqFfea0dXdd9vqai=hGuQ8kuc9pgc9s8qqaq=dirpe0xb9q8qiLsFr0=vr0=vr0dc8meaabaqaciaacaGaaeqabaqabeGadaaakeaacuWGmbatgaqcaaaa@2DDD@(*h*), of the estimated *K *function K^
 MathType@MTEF@5@5@+=feaafiart1ev1aaatCvAUfKttLearuWrP9MDH5MBPbIqV92AaeXatLxBI9gBaebbnrfifHhDYfgasaacH8akY=wiFfYdH8Gipec8Eeeu0xXdbba9frFj0=OqFfea0dXdd9vqai=hGuQ8kuc9pgc9s8qqaq=dirpe0xb9q8qiLsFr0=vr0=vr0dc8meaabaqaciaacaGaaeqabaqabeGadaaakeaacuWGlbWsgaqcaaaa@2DDB@(*h*) that, when plotted on the y axis as L^
 MathType@MTEF@5@5@+=feaafiart1ev1aaatCvAUfKttLearuWrP9MDH5MBPbIqV92AaeXatLxBI9gBaebbnrfifHhDYfgasaacH8akY=wiFfYdH8Gipec8Eeeu0xXdbba9frFj0=OqFfea0dXdd9vqai=hGuQ8kuc9pgc9s8qqaq=dirpe0xb9q8qiLsFr0=vr0=vr0dc8meaabaqaciaacaGaaeqabaqabeGadaaakeaacuWGmbatgaqcaaaa@2DDD@(*h*) - (*h*), aids in the visual inspection of the *K *function over a range of distances. Besag [43] recommended the transformation of L^
 MathType@MTEF@5@5@+=feaafiart1ev1aaatCvAUfKttLearuWrP9MDH5MBPbIqV92AaeXatLxBI9gBaebbnrfifHhDYfgasaacH8akY=wiFfYdH8Gipec8Eeeu0xXdbba9frFj0=OqFfea0dXdd9vqai=hGuQ8kuc9pgc9s8qqaq=dirpe0xb9q8qiLsFr0=vr0=vr0dc8meaabaqaciaacaGaaeqabaqabeGadaaakeaacuWGmbatgaqcaaaa@2DDD@(*h*) = [K^
 MathType@MTEF@5@5@+=feaafiart1ev1aaatCvAUfKttLearuWrP9MDH5MBPbIqV92AaeXatLxBI9gBaebbnrfifHhDYfgasaacH8akY=wiFfYdH8Gipec8Eeeu0xXdbba9frFj0=OqFfea0dXdd9vqai=hGuQ8kuc9pgc9s8qqaq=dirpe0xb9q8qiLsFr0=vr0=vr0dc8meaabaqaciaacaGaaeqabaqabeGadaaakeaacuWGlbWsgaqcaaaa@2DDB@_*e*_(*h*)/*π*]^1/2^. The K^
 MathType@MTEF@5@5@+=feaafiart1ev1aaatCvAUfKttLearuWrP9MDH5MBPbIqV92AaeXatLxBI9gBaebbnrfifHhDYfgasaacH8akY=wiFfYdH8Gipec8Eeeu0xXdbba9frFj0=OqFfea0dXdd9vqai=hGuQ8kuc9pgc9s8qqaq=dirpe0xb9q8qiLsFr0=vr0=vr0dc8meaabaqaciaacaGaaeqabaqabeGadaaakeaacuWGlbWsgaqcaaaa@2DDB@_*e *_is the edge-corrected *K *function estimate defined by Ripley [44] as K^e(h)=λ^−1∑i=1N∑j=1,j≠iNwijδ(dij<h)
 MathType@MTEF@5@5@+=feaafiart1ev1aaatCvAUfKttLearuWrP9MDH5MBPbIqV92AaeXatLxBI9gBaebbnrfifHhDYfgasaacH8akY=wiFfYdH8Gipec8Eeeu0xXdbba9frFj0=OqFfea0dXdd9vqai=hGuQ8kuc9pgc9s8qqaq=dirpe0xb9q8qiLsFr0=vr0=vr0dc8meaabaqaciaacaGaaeqabaqabeGadaaakeaacuWGlbWsgaqcamaaBaaaleaacqWGLbqzaeqaaOGaeiikaGIaemiAaGMaeiykaKIaeyypa0dcciGaf83UdWMbaKaadaahaaWcbeqaaiabgkHiTiabigdaXaaakmaaqahabaWaaabCaeaacqWG3bWDdaWgaaWcbaGaemyAaKMaemOAaOgabeaakiab=r7aKjabcIcaOiabdsgaKnaaBaaaleaacqWGPbqAcqWGQbGAaeqaaOGaeyipaWJaemiAaGMaeiykaKcaleaacqWGQbGAcqGH9aqpcqaIXaqmcqGGSaalcqWGQbGAcqGHGjsUcqWGPbqAaeaacqWGobGta0GaeyyeIuoaaSqaaiabdMgaPjabg2da9iabigdaXaqaaiabd6eaobqdcqGHris5aaaa@5878@, where the weight *w*_*ij *_is the proportion of the circumference of the event-centered circle with radius *d*_*ij *_that is within the study area and λ^
 MathType@MTEF@5@5@+=feaafiart1ev1aaatCvAUfKttLearuWrP9MDH5MBPbIqV92AaeXatLxBI9gBaebbnrfifHhDYfgasaacH8akY=wiFfYdH8Gipec8Eeeu0xXdbba9frFj0=OqFfea0dXdd9vqai=hGuQ8kuc9pgc9s8qqaq=dirpe0xb9q8qiLsFr0=vr0=vr0dc8meaabaqaciaacaGaaeqabaqabeGadaaakeaaiiGacuWF7oaBgaqcaaaa@2E77@ is the intensity estimate, equal to the number of events in the study area divided by the area of the study. The expected value under complete spatial randomness (CSR) of L^
 MathType@MTEF@5@5@+=feaafiart1ev1aaatCvAUfKttLearuWrP9MDH5MBPbIqV92AaeXatLxBI9gBaebbnrfifHhDYfgasaacH8akY=wiFfYdH8Gipec8Eeeu0xXdbba9frFj0=OqFfea0dXdd9vqai=hGuQ8kuc9pgc9s8qqaq=dirpe0xb9q8qiLsFr0=vr0=vr0dc8meaabaqaciaacaGaaeqabaqabeGadaaakeaacuWGmbatgaqcaaaa@2DDD@(*h*) - *h *is close to zero. The plot in the top of Figure 2 is of L^
 MathType@MTEF@5@5@+=feaafiart1ev1aaatCvAUfKttLearuWrP9MDH5MBPbIqV92AaeXatLxBI9gBaebbnrfifHhDYfgasaacH8akY=wiFfYdH8Gipec8Eeeu0xXdbba9frFj0=OqFfea0dXdd9vqai=hGuQ8kuc9pgc9s8qqaq=dirpe0xb9q8qiLsFr0=vr0=vr0dc8meaabaqaciaacaGaaeqabaqabeGadaaakeaacuWGmbatgaqcaaaa@2DDD@(*h*) - *h *for cases evaluated at a range of distances over the study area with 999 Monte Carlo simulations of CSR to create 95% confidence bands to assess significance of deviations in the transformed, estimated *K *function from CSR. Note that the data points in the study are projected to Universal Transverse Mercator (UTM), zone 17 coordinates with meters as the distance unit. The plot indicates that there is significant clustering at smaller distances (less than 100,000 meters) and generally insignificant clustering at intermediate and large distances, where the transformed *K *function falls within the confidence bands. While the finding of clustering in the cases may seem significant, it is not the complete story of this phenomenon. To see this, we must inspect the bottom plot in Figure 2, which is a plot of L^
 MathType@MTEF@5@5@+=feaafiart1ev1aaatCvAUfKttLearuWrP9MDH5MBPbIqV92AaeXatLxBI9gBaebbnrfifHhDYfgasaacH8akY=wiFfYdH8Gipec8Eeeu0xXdbba9frFj0=OqFfea0dXdd9vqai=hGuQ8kuc9pgc9s8qqaq=dirpe0xb9q8qiLsFr0=vr0=vr0dc8meaabaqaciaacaGaaeqabaqabeGadaaakeaacuWGmbatgaqcaaaa@2DDD@(*h*) - *h *for the controls. The figures shows a similar pattern for cases and controls, which indicates that the significant clustering at smaller distances for cases is due to clustering in the underlying population and not clustering in the cases above what is observed in the at-risk population. While a visual comparison of the *K *functions for cases and controls shows no clear differences between the two, a test of difference in *K *functions is needed to definitively answer the inquiry of potential clustering in childhood leukemia.

**Figure 3:**
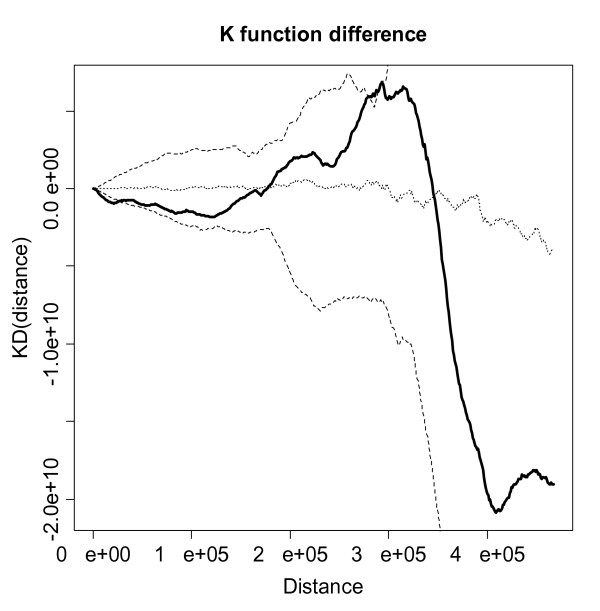
Difference in case and control *K *functions with confidence bands (dashed) and distance in meters.

Fortunately, when using the *K *function, one can calculate a difference of *K *functions for cases and controls to detect differences in patterns in the two point processes. The simple formula for doing so is *KD*(*h*) = *K*_*cases*_(*h*) - *K*_*controls*_(*h*). For this difference in cases and controls, one can calculate confidence bands using Monte Carlo randomization to evaluate significance of any differences in patterning. To do so, one first conditions on the locations of cases and controls, randomizes the case labels among the locations, and then calculates the test statistic *KD*(*h*) at a range of distances. This procedure is performed a set number of times and the test statistic from the original data is compared to the upper 97.5% limit of the test statistic values from the Monte Carlo randomizations to assess significance. Figure 3 is a plot of the function *KD*(*h*) over a range of distances for 999 randomizations of the case labels and shows that, overall, there are not significant differences in the *K *functions for cases and controls, as the line for *KD*(*h*) falls mostly within the 95% confidence bands. The key area of interest in the plot as in indication of clustering is the area above the 95% confidence band, primarily at smaller distances based on intuitive properties of a cluster. If the *KD*(*h*) line was in this area, it would indicate significant clustering. That is clearly not the case with these data. Therefore, the statistical test of difference in *K *functions for cases and controls verifies the visual impression drawn from Figure 2 of no clustering in cases that is different than that in controls. The *K *function difference plot in Figure 3 was made using R software, as ClusterSeer software currently produces only individual case and control *K *functions.

### Kernel intensity function

**Figure 4:**
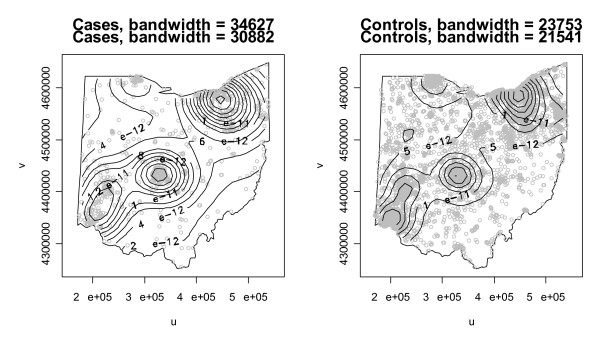
Contours of estimated kernel density functions for cases and controls with UTM coordinates.

While the *K *function is designed to test for clustering, the kernel intensity function introduced by Kelsall and Diggle [45] can be used to test for clustering and the presence and location of local clusters. In fact, it is the only test in this comparison that can explicitly evaluate both conditions. The kernel intensity function calculates the number of events expected in an area at location *s *(intensity) or the probability of an event occurring at location *s *(density) using a kernel function. The intensity and density functions are proportional and are often used interchangeably in practice [19]. The kernel function requires a bandwidth that determines the size of the kernel and the overall smoothness of the resulting estimate. In a Gaussian kernel, which we make use of in this study, the bandwidth corresponds to the standard deviation and larger bandwidths result in smoother kernel intensity functions. We use Scott's [46] rule for optimal bandwidth selection in a Gaussian kernel, where Scott's rule considers the number of events and spatial variance of events in a point pattern when calculating the bandwidth. The two-dimensional Gaussian kernel we use has a bandwidth in both the *u *and *v *directions, where the map coordinates are in the form of (*u*, *v*). Applying Scott's rule to the Ohio data results in bandwidths of 34,627 meters in the *u *direction and 30,882 meters in the *v *direction for cases and bandwidths of 23,753 meters in the *u *direction and meters units in the *v *direction for controls. The kernel function uses distance between a location *s *and all other points as input to calculate an intensity function at *s*. We evaluate the kernel function at each point on a 40 × 40 grid that completely contains the study area, where the distance between adjacent grid points is approximately 11,619 meters. Figure 4 contains contour plots of the kernel density function for cases and controls separately. The plots show similar patterns in the probability of an event occurring at a given point in the study area, where the probabilities are highest in the three largest metropolitan areas of Cincinnati, Columbus, and Cleveland. While the plots are somewhat informative, a formal test of difference in the patterns would be helpful.

**Figure 5:**
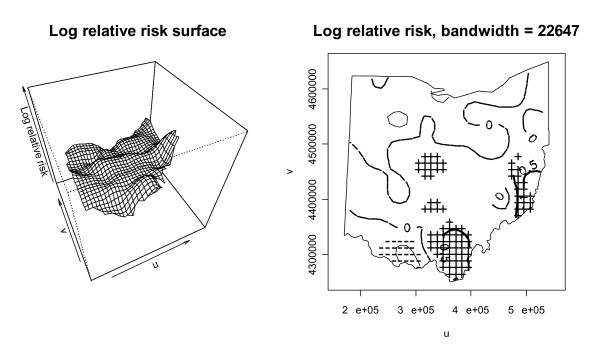
**Log relative risk surface using kernel density functions with kernel bandwidth = 22,647 meters**. The significant risk areas according to Monte Carlo simulation are indicated on the right plot using "-" for points below the 2.5% simulation value and "+" for points above the 97.5% value. The contour lines on this plot indicate the log relative risk.

**Figure 6:**
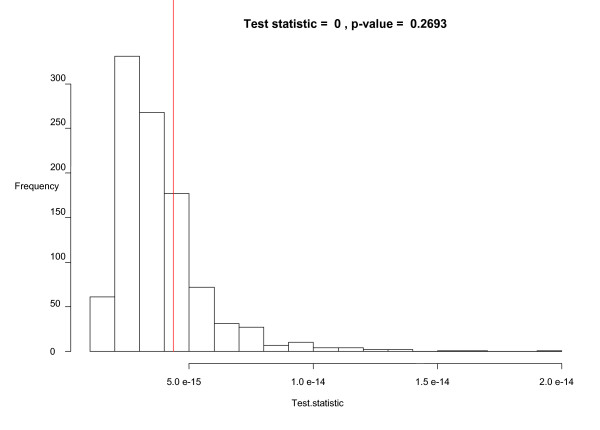
Simulated values for the test of global clustering using kernel density functions (p-value = 0.27).

**Figure 7:**
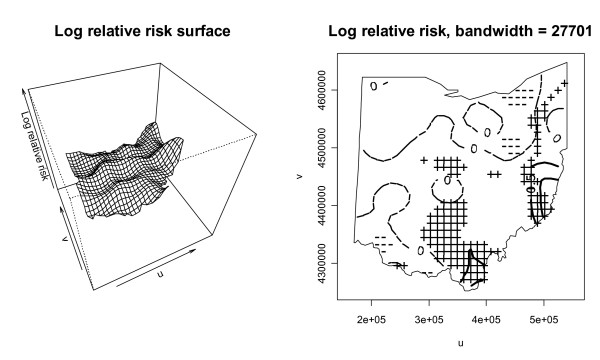
Log relative risk surface using kernel density functions with kernel bandwidth = 27,701 meters.

Conveniently, one can calculate a log ratio of kernel intensity functions for cases and controls to get a log relative risk at a location on the grid. When considering all grid points that cover the study area, this yields a log relative risk surface. To calculate this log relative risk surface, we first redefine the kernel bandwidth with the kernel intensity function ratio because it is beneficial to have the same kernel bandwidth in both cases and controls in order to have an equal spatial extent covered in the numerator and denominator of the ratio. We initially choose for a kernel bandwidth in both dimensions the mean of the control optimal bandwidths calculated previously, which is 22,647 distance units. We favor the controls in this bandwidth selection because there are many more of them than cases and they should in theory reflect the underlying population distribution. This bandwidth yields a smaller kernel than with the cases, and will reveal more detail in the estimated kernel intensity function but will also be more variable. With the kernel intensity function ratio, one can again use Monte Carlo randomization of the case labels to detect significant local differences in case and control intensities. Figure 5 shows the log relative risk surface using the log ratio of kernel density functions for cases and controls and also shows the significant areas of log relative risk using the 2.5% lower and 97.5% upper tolerance limits from 999 Monte Carlo randomizations of the case labels. The hatched ("+") areas in the plot on the right indicate significant local clusters of elevated log relative risk and correspond to the higher points on the log relative risk surface map. The areas with "-" symbols have significantly low disease incidence. The contour lines on this plot correspond to smoothed levels of log relative risk. The contour lines with value 0.5 indicate relative risks of approximately 1.6 and the contour line of 1 signifies relative risks of approximately 2.7. The highest log relative risk is just over 1.5 (relative risk of 4.5) and is found in the hatched area in eastern Ohio. The plots suggest that there are areas of higher disease incidence in central, southern, and eastern Ohio, and also an area of lower incidence southeast of Cincinnati. As mentioned earlier, one can also test for overall clustering with the kernel intensity function method using a mathematical summary of the local function ratios. The test statistic is a sum of squared log ratios of kernel intensity functions across the study area. Monte Carlo randomization is used to assess significance of the test statistic for clustering. Figure 6 is a histogram of the values of the test statistic from the Monte Carlo randomizations of the case labels, along with the test statistic for the original data plotted on the histogram as a vertical line. The p-value of 0.27 indicates that there is no significant global clustering in the cancer cases, considering the distribution of the at-risk population. To explore the sensitivity of the results to the selected kernel bandwidth, we next choose a compromise kernel bandwidth in both kernel dimensions as the mean of the optimal case and control bandwidths calculated previously, which results in a bandwidth of 27,701 distance units. The log relative risk surface and significant risk areas with this new kernel bandwidth are plotted in Figure 7. The bandwidth used to generate Figure 7 is larger than the one used to generate Figure 5, and the new resulting risk surface is slightly smoother than the one in Figure 5. The areas of significant elevated risk visible in Figure 5 are also present in Figure 7, but the larger bandwidth extends the significant cluster areas and now two north-south swaths are clearly apparent. The largest log relative risk with this bandwidth is approximately 1.4 (relative risk 4.4) and is located in the same hatched area in eastern Ohio as in Figure 5. As was the case with the first bandwidth, the test for overall clustering using the summary of the local log ratios of kernel functions with the second bandwidth is not significant, but the p-value decreases to 0.08 in this case.

### Cuzick and Edwards' method

**Figure 8:**
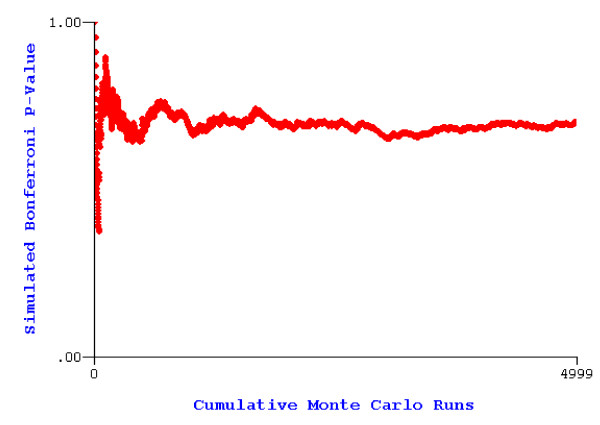
Overall Bonferroni p-value for Cuzick and Edwards' method versus number of Monte Carlo randomizations.

Similar to the *K *function, Cuzick and Edwards' method [39] tests for clustering in a point pattern. It measures the tendency of a point process to cluster at certain specified numbers of nearest neighbors and asks if there are more cases then expected under random labeling in the *k *locations nearest each case. Cuzick and Edwards' method counts the number of cases within *k *nearest case and control neighbors of each case and sums these counts to make one test statistic *T*(*k*) for each *k*. In practice, this method requires specification of the *k *nearest neighbors in advance, and, typically, one would specify a range of *k *nearest neighbors to use. In this case, there is an adjustment of the overall p-value, using both the Bonferroni and Simes adjustments, to reflect the multiple nearest neighbor tests. The Bonferroni adjustment is *p*_*B *_= *n*·*min*[*p*_*i*_] and the Simes adjustment is *p*_*S *_= *min*[(*n*-*i*+1)·*p*_*i*_], where *n *is the number of tests, *p*_*i *_is the p-value for the *i*th test, and *i *is the test index, which is sequential from lowest to highest p-value for the Simes adjustment [33]. ClusterSeer software uses Monte Carlo randomization of the case labels among the given locations and also a normal approximation to evaluate significance of each nearest neighbor test statistic [33]. Some of the results of applying Cuzick and Edwards' method to all leukemia cases in the dataset are listed in Table 1. We specified 10 nearest neighbor tests, using *k *from 1 to 10 and used 4999 Monte Carlo randomizations to evaluate the overall p-value. As listed in the table, neither the normal approximation nor the Monte Carlo p-values indicate significant tests for the ten levels of nearest neighbors. The tests for *k *= 6 and *k *= 7 are somewhat close to significant (at the 0.05 level) with the Monte Carlo randomization assessment and *k *= 7 and *k *= 8 are also close to significant with the normal approximation. The overall Bonferroni and Simes p-values for the normal approximation are 0.73 and 0.22, respectively, where the Bonferroni is overly conservative and Simes is less conservative. The overall Bonferroni and Simes p-values for the Monte Carlo randomizations are 0.70 and 0.17, respectively. These values indicate that there is no clustering of cases among nearest neighbors in all of the leukemia cases. Figure 8 is a plot of the overall Bonferroni p-value over the runs of the Monte Carlo randomizations of the case labels. The plot shows that the p-value has stabilized over the runs and is trustworthy for testing.

**Table 1:** Results of the Cuzick and Edwards' test

k	T[k]	E[T]	Var[T]	z	Upper-tail P-value	Monte Carlo P-value
1	70	67.66	92.96	0.24	0.40	0.41
2	137	135.32	194.01	0.12	0.45	0.54
3	210	202.98	296.67	0.41	0.34	0.26
4	278	270.63	400.36	0.37	0.36	0.51
5	352	338.29	505.31	0.61	0.27	0.23
6	432	405.95	611.51	1.05	0.15	0.07
7	512	473.61	718.28	1.43	0.08	0.07
8	583	541.27	826.37	1.45	0.07	0.36
9	638	608.93	2432.11	0.59	0.28	0.95
10	714	676.58	2541.57	0.74	0.23	0.17

The table contains the test statistic *T*(*k*), the expected test value and variance using the normal approximation, and the normal approximation and Monte Carlo randomization p-values for each *k *nearest neighbors

We next applied the Cuzick and Edwards method to subsets of the case data, using three sets for ages 0–4, 5–9, and 10–14 and one for ALL type cases. In the interest of space, we report only the summary of each subset analysis. There was no overall significant clustering or significant clustering at any level of *k *for cases age 0–4. There was significant clustering for cases age 5–9 with *k *= 7 (p-value = 0.04), but no overall significant clustering. There was no overall significant clustering or significant clustering for cases age 10–14. There was significant clustering for cases of type ALL with all ages with *k *= 6 (p-value = 0.048), but no overall significant clustering. The results suggest some clustering at six or seven nearest neighbors, depending on the subset of cases, but no overall clustering, regardless of the set of cases. The relevance of nearest neighborhood structures of size six or seven for some leukemia cases is unknown at this point in time, but could be a subject of future inquiry with a credible hypothesis. However, there may not be a factor that can be quantified to explain the significance of this apparent structure.

### SaTScan

**Figure 9:**
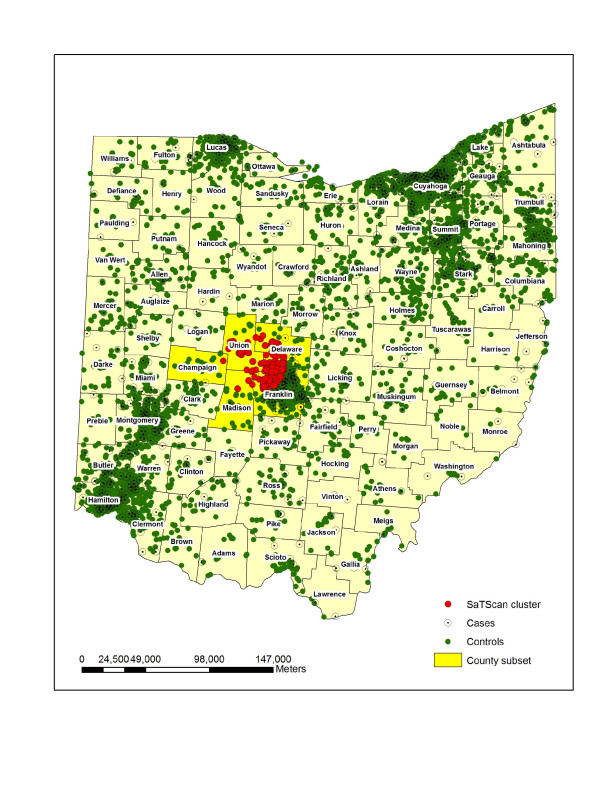
Most likely SaTScan cluster for all cases (43 cases, p-value 0.81).

Kulldorff's scan statistic [47] as implemented in SaTScan software is explicitly a test for clusters, as noted in [1,33,34,48]. For case-control data, it calculates local rates inside scanning circles of various sizes using the Bernoulli model, where cases are designated as ones and controls are designated by zeros. SaTScan places circles at each case and control, ranging in radius from the smallest inter-event distance to typically the distance that contains half the population in the study area, and calculates a likelihood ratio test of each potential cluster, where the likelihood ratio test compares the alternative hypothesis that there is an increased risk of disease inside the circle with the null hypothesis that the disease risk is the same inside and outside the circle. The circle with the maximum likelihood is the most likely cluster. SaTScan calculates the p-value of the most likely cluster using the likelihood ratio test and repeated Monte Carlo randomizations of the case labels. The rank of the most likely likelihood ratio test among all randomization tests determines the p-value. As output, SaTScan reports the most likely cluster and secondary clusters, along with the corresponding significance values. The scan statistic in SaTScan has been applied to Poisson distributed count data [1,49], in addition to Bernoulli case-control data [19]. We applied Kulldorff's scan statistic in SaTScan to all of the cases and then the same four case subsets described in the Cuzick and Edwards' method section. The most likely cluster found by SaTScan using all of the cases is displayed in Figure 9. This particular cluster contains 43 cases and is located northwest of the city of Columbus in central Ohio. The p-value of 0.81 for this potential cluster indicates that it is not statistically significant. The most likely cluster for ALL type cases is of size 12, has a p-value of 0.73, and is located in southern Ohio. The most likely cluster for cases age 0–4 has 10 cases, a p-value of 0.71, and is located in southwest Ohio, northeast of the city of Cincinnati. The most likely cluster for cases age 5–9 has 23 cases, a p-value of 0.56 and is located in northeast Ohio, south of the city of Cleveland. The most likely cluster for cases age 10–14 is comprised of three cases, has a p-value of 0.33, and is located in Union County, in part of the most likely cluster found with all cases. Based on the p-values from the individual likelihood ratio tests, none of the most likely clusters found by SaTScan are statistically significant.

Typically, when public health professionals investigate a potential cluster, they use a much smaller study area than a state, perhaps using the spatial extent of a county or area surrounding a town. To better mimic this type of investigation, and to evaluate the sensitivity of the spatial scan statistic's test for significance to the size of the study area, we next report results from a cluster detection analysis in a spatial subset of the study area. We selected a contiguous set of five counties, Union, Franklin, Delaware, Madison, Champaign, which contained the most likely SaTScan cluster for cases age 0–14. In practice, a public health analyst would not refine the study area around a previously detected cluster. The most likely cluster found by SaTScan with this subset of data is the same 43 cases in the most likely cluster with all of the Ohio data, but the p-value is now 0.71, instead of the value of 0.81 found with the complete dataset. The highlighted subset of counties and most likely cluster are visualized in Figure 9. This raises a point that the size of the study area can impact the result of the significance test in SaTScan. Naturally, the conclusion of no significant cluster in this situation does not change, but it could in some circumstances, with a cluster changing status from insignificant to significant depending on how the analyst defines the study area. We make note of this as more of a practical issue for consideration then as a criticism of SaTScan. Since the study area provides the context for interpretation in the investigation of the question of whether cases cluster in an area, the question of interest changes if the study area is changed. The relationship between study area size and the research question considered in a cluster detection study has also been discussed by Jacquez and Greiling [50].

## Discussion

The three methods used to detect global clustering, the *K *function, the kernel intensity function ratio summary, and Cuzick and Edwards' method, all found no statistically significant clustering of childhood (age 0–14) leukemia in Ohio from 1996–2003. Cuzick and Edwards' method also found no significant clustering in three separate age groups of cases and ALL type cases. These findings are not entirely surprising given the large and diverse study area of Ohio, in which it is doubtful that one particular risk factor would have a consistent or sustained effect across space that would result in clustering demonstrated at the state scale. It is more likely that factors which could explain clustering of cases would have local or regional influence, and one factor could be associated with clustering in one area while another factor could be related to clustering in a different area. Given the scale of the study area in this analysis, the search for local cancer clusters is the more useful investigation, and also the one with more public interest. In investigation of potential clusters, there were inconsistent findings from the two methods used to detect clusters. The kernel intensity function ratio suggested some significant local clusters in cases age 0–14 in portions of central and eastern Ohio, while the spatial scan statistic in SaTScan found no significant clusters. SaTScan also found no significant clusters for three different age groups and ALL type cases. Some reassurance comes from the fact that some of the most likely SaTScan clusters are in the same areas as the significant elevated log relative risk areas from the kernel intensity function ratios. Still, the cancer cluster investigator is left to wonder which results are more trustworthy in this circumstance. Unfortunately, without a well-designed simulation study that reflects the current study situation and where the true clusters are known, one cannot definitively reach a conclusion on this matter. A simulation study that tests for different types of clusters is left for future research.

One practical reason to favor the kernel intensity function method is that it tests for local clusters and explicitly uses a summary measure of the local results to test for global clustering; it is unique in this regard. Another advantage of the kernel intensity function method is that it provides the log relative risk surface over the entire study area, so one can visualize the local peaks and valleys in the risk of disease. In addition, the kernel is more flexible in its shape than SaTScan's circular scanning window. There have been advancements in the literature, however, with scan statistics designed to detect elliptical clusters [51] as well as more flexibly shaped clusters [52]. An arbitrary shaped non-scanning method based on minimum spanning trees has also been recently introduced [53]. A disadvantage with the kernel intensity ratio is that one must select the bandwidth in advance of calculating the log relative risk, and results can certainly vary depending on the selected bandwidth. One possibility to overcome this is may be to use a Bayesian framework for kernel intensity estimation [54], where the kernel bandwidth would be estimated from the data while simultaneously calculating the log relative risks.

Numerous practical issues with spatial case-control cluster detection were encountered in this study. First, the selection of controls is crucial in these case-control spatial clustering studies. We found a traditional epidemiology ratio of 3 to 1 to be inadequate with our systematic sampling scheme, and believe that would be true with a purely random sampling scheme as well. We tentatively recommend using as many controls as possible taking into consideration the cost in acquiring them and in computing, as some methods such as the *K *function and SaTScan can take substantial run time with a large number of points in the study. More research is needed to determine, if possible, an optimal number of controls and sampling scheme. In this study, we also realized the importance of avoiding unnecessary spatial error when possible, in terms of geocoding and map units. Of course, there is inherent locational uncertainty in these data [55]. Invariably, in the address matching process of individual records there will be observations for which an exact address match is not possible. These records can be geocoded to census boundary or ZIP Code centroids or omitted from the study, where the decision on the handling of these records could depend on the study area scale. For a large study area, using census tract or ZIP Code centroids matches may be deemed acceptable in searching for an approximate cluster location, where county centroids may be viewed as providing spatial locations that are too inaccurate. We omitted centroid-matched points after checking visually that they were not spatially influential, i.e. occurring in one area only or exclusively in rural areas, to avoid inducing artificial clustering in cases or controls. We also used UTM map coordinates to prevent adding spatial error to our Euclidean distance calculations. An alternative would be to use great circle distance calculation for records in latitude and longitude coordinates.

## Conclusion

This comparative study for childhood leukemia clustering and clusters in Ohio is the first one with individual level case and control data. The study produced results that lead to different conclusions based on the method utilized regarding the significance of clusters and also revealed several open research issues in practical spatial cluster detection. In summary, we found some evidence, although inconclusive, of significant local clusters in childhood (age 0–14) leukemia in Ohio during years 1996–2003, but no significant overall clustering when considering all case ages simultaneously. The spatial scan statistic in SaTScan found no significant clusters, while the kernel intensity function ratio found clusters, some of irregular shape, in areas of central, southern, and eastern Ohio. It should be pointed out that different methods used to test for clustering look for different types of clusters, and one method may not find a cluster while another method does, and both may be correct depending on the underlying true cluster. Consideration of the potential shape of clusters in the study area appears to be an important issue. In considering future work with these data, a subsequent study should test for spatial clusters in ALL type cases by age groups based on the finding of Dockerty and his coauthors [3] of significant clustering using Cuzick and Edwards' method in age subgroups of ALL cases, but not in ALL cases age 0–14. Additional future work could systematically investigate the sensitivity of the results from the methods selected to the ratio of controls to cases, to different sizes of the study area, and to different control sampling schemes, such as simple random, stratified, or probability proportional to size cluster sampling. A potentially interesting and relevant future comparison would be between the results presented here to those from methods for regional count data at the county level. There is additional effort involved in spatial case-control cluster studies compared to regional count cluster studies, and it would be worthwhile to see if the additional data needs and computational cost result in substantially increased power to detect clusters.

## Competing interests

The author(s) declare that they have no competing interests.

## Authors' contributions

DW designed the study, performed the analysis, and drafted the manuscript.
